# Reducing Both Pgp Overexpression and Drug Efflux with Anti-Cancer Gold-Paclitaxel Nanoconjugates

**DOI:** 10.1371/journal.pone.0160042

**Published:** 2016-07-28

**Authors:** Fei Li, Xiaofei Zhou, Hongyu Zhou, Jianbo Jia, Liwen Li, Shumei Zhai, Bing Yan

**Affiliations:** School of Chemistry and Chemical Engineering, Shandong University, Jinan, 250100, China; University of Helsinki, FINLAND

## Abstract

Repeated administrations of anti-cancer drugs to patients often induce drug resistance. P-glycoprotein (Pgp) facilitates an efficient drug efflux, preventing cellular accumulation of drugs and causing multi-drug resistance (MDR). In this study, we developed a gold-paclitaxel nanoconjugate system to overcome MDR. Gold nanoparticles (GNPs) were conjugated with β-cyclodextrin enclosing paclitaxel (PTX) molecules and PEG molecules. GNP conjugates were effectively endocytosed by both drug-sensitive human lung cancer H460 cells and Pgp-overexpressed drug-resistant H460_PTX_ cells. Compared with PTX, PGNPs did not induce the Pgp overexpression in drug-sensitive H460 cells after long-term treatment and also avoided being pumped out of cells by overexpressed Pgp molecules in H460_PTX_ with a 17-fold lower EC_50_ compared to PTX. Fluorescent microscopy and flow cytometry further confirmed that fluorescent labeled PGNPs (f-PGNPs) maintained a high cellular PTX level in both H460 and H460_PTX_ cells. These results demonstrated that nano-drug conjugates were able to avoid the development of drug resistance in sensitive cells and evade Pgp-mediated drug resistance and to maintain a high cytotoxicity in drug-resistant cancer cells. These findings exemplify a powerful nanotechnological approach to the long-lasting issue of chemotherapy-induced drug resistance.

## Introduction

In cancer chemotherapy, repeated administrations of anti-cancer drugs often induce drug resistance and lead to treatment failure in patients [1, 2]. For example, many effective anti-cancer drugs, such as doxorubicin, vincristine, actinomycin-D, and paclitaxel (PTX), could induce the multi-drug resistance (MDR) [3], a phenotype of cross-resistance to multiple drugs with both similar and unrelated structures. Although MDR can be caused by various mechanisms, the overexpression of transporter proteins that pump drugs out of cells is the major mechanism of MDR [4]. Pgp is one such protein, serves as a membrane pump, binds drugs with diverse chemical structures and pump them out of the drug resistant cancer cells [5–7]. Furthermore, previous investigations have shown that a high drug concentration is a prerequisite for triggering MDR gene expression in drug-sensitive cancer cells [8, 9]. Due to these two obstacles in chemotherapy, effective cancer treatment has been severely hindered. Therefore, it would be desirable to develop chemotherapeutics to both avoid Pgp overexpression and reduce drug efflux in order to increase the efficacy of anti-cancer drugs.

In the past few years, accumulating evidence showed that nanotechnology has the largest impact on medicine when solving tough problems for which conventional protocols fail. To date, nanocarriers have been explored for a variety of applications such as cancer diagnosis [10, 11], drug delivery [12], imaging [13], photothermal ablation of tumours [14–17] and radiation sensitizers [18, 19], offering unique advantages over free drugs [20]. In particular, drug administration by nanocarriers displayed great perspective in the strategies considered to overcome MDR through changing the internalization pathways and/or intracellular release style of drugs, suppressing the activity of the MDR efflux pump, or inhibiting the expression of genes responsible for the activity of efflux pumps, detoxification and apoptosis [21–27]. Among all the drug nanocarriers that have been reported, gold nanoparticles (GNPs) possess excellent characteristics, such as precisely-controlled size, tunable optical properties, robust stability, biocompatibility and diversified postsynthetic surface modification, which enable their promising use as one of the best drug nanocarriers [28–31]. For examples, PEGylated GNPs binding with recombinant human tumor necrosis factor alpha (TNF-α) showed potential use in targeting solid tumors in advanced stage cancer patients [32]. GNPs covered with cyclodextrin were useful in antitumor drugs delivery for therapeutic purposes [33–37]. Recently, GNPs conjugated with anticancer drugs have shown great promise in overcoming MDR [27]. For instance, doxorubicin grafted-PEGylated GNPs overcome MDR in Doxorubicin-selected P-gp-overexpressing cancer cells [38]. DOX-tethered GNPs could significantly overcome P-gp-mediated drug resistance by a combination of enhanced doxorubicin cellular entry and a responsive intracellular release of doxorubicin in acidic organelles [23]. GNPs loaded with PTX molecules through DNA linkers increased drug efficacy in Paclitaxel-resistant cell lines [39].

Based on those progresses [20, 21, 23, 25, 38, 39], we hypothesize that drug molecules associated with a nanocarrier would be released slowly in cancer cells, avoiding activation of drug resistance-related genes. Morever, nanoparticles are also expected to be a poor substrate for Pgp [40, 41], allowing drugs to remain inside the cells, where their anti-cancer activities are most effective. Therefore, with a single nanoconjugate design, we may be able to achieve two important goals simultaneously. In this study, we designed and prepared NP-drug nanoconjugates by loading paclitaxel on GNPs modified with β-cyclodextrin (β-CD) and PEG_5000_. The gold-paclitaxel nanoconjugates could overcome MDR through simultaneously preventing the overexpression of Pgp proteins in drug-sensitive cells and evading Pgp-induced drug efflux to achieve potent cancer cell death in drug-resistant cells.

## Materials and Methods

### Reagents and cell line

Unless otherwise indicated, all chemical reagents were obtained from Sigma-Aldrich (St. Louis, MO, USA) and used without further purification. RPMI 1640, fetal bovine serum, penicillin, streptomycin, and all other tissue culture reagents were obtained from Life Technologies (Grand island, NY, USA). Pgp low-expressed non-small cell lung cancer (NSCLC) cell line H460 was provided by Dr. Bingliang Fang (The University of Texas, MD Anderson cancer center, USA). The cells were maintained in RPMI-1640 medium supplemented with 10% fetal bovine serum and 1% penicillin-streptomycin. Cells were cultured in a humidified incubator at 37°C with 5% CO_2_. In all experiments, cell line was used before passage 40.

### Preparation of PGNPs

GNPs with 15 nm diameter and sulfhydryl group modified cyclodextrin (SH-β-CD) were synthesised according to our previous work [42]. 3 mg of NH_2_-PEG_5000_-SH was then added to 300 mL of the above GNPs solution and stirring for 24 hrs. 300 uL of SH-β-CD solution was added to the mixture and stirred for another 24 hrs. The free sodium citrate, NH_2_-PEG_5000_-SH, and SH-β-CD were removed by six times washing (ultrapure water, 40 mL × 6) using regular centrifugation (50,000 g) at 4°C for 30 min. The retained nanoparticles were re-suspended in ultrapure water and the gold content was detected using ICP-AES (Optima 7000DV, Perkin Elmer, Inc., United States).

To obtain paclitaxel loaded GNPs (PGNPs), 300 μL of PTX-DMSO solution (0.4 mM) was added into 30 mL of the above GNP solutions. After stirring for 48 hrs at 4°C, the result solutions was allowed to go through syringe driven filter unit (0.22 μm, Millex®GP) to form a sterile PGNPs stock solution.

### Determination of PTX loading and releasing

At different time points during the load reaction, 3 mL of the reaction solution was taken out, centrifuged at 50,000 g for 30 min and washed with PBS (5 mL × 2). The supernatant was extracted with EtOAc (10 mL× 6). The organic phase was dried with anhydrous MgSO_4_ and concentrated to dryness under vacuum at room temperature. Then the retained sediment was re-dissolved in 30 μL DMSO and the concentration of PTX was determined by HPLC (PTX standard solutions: 200, 100, 50, 25, 10, 5, 2, 1 μM in DMSO). The detailed HPLC elution conditions were as follows: Waters Xterra MS-C18 column (5 μm, 2.1 mm × 50 mm); The mobile phases were acetonitrile and water; Gradient elution condition: 0 min, 25% acetonitrile; 1 min, 40% acetonitrile; 2.0 min, 100% acetonitrile; 6.0 min, 100% acetonitrile; 7.0 min, 70% acetonitrile; 8.0 min, 70% acetonitrile, and 10 min, 25% acetonitrile. The flow rate was 0.3 mL/min. Detection wavelength: 254 nm; Column temperature: 25°C.

The release of PTX from PGNPs was determined by a similar method. 10 mL PGNPs stock solution was centrifuged at 50,000 g for 30 min, washed with PBS for three times and then re-suspended in 10 mL PBS (pH = 7.5 and pH = 5.5). Then the suspensions were incubated at 37°C. At different time point, the suspensions were centrifuged at 50,000 g for 30 min and the sediments were washed with PBS and re-suspended in PBS of different pH for further release experiment. The PTX concentration in the obtained supernatant was also determined by HPLC.

To determine the number of FITC-PTX per PGNP, 10 mL f-PGNPs stock solution was centrifuged at 50,000 g for 30 min and washed with ultrapure water (5 mL × 3). The FITC-PTX in the obtained supernatant was determined using fluorescent spectrophotometer (F4500, Hitech, Japan) (FPTX standard solutions: 200, 100, 50, 25, 10, 5, 2, 1 μM in ultrapure water with 0.4‰ DMSO).

### Hydrodynamic size and zeta potential measurement

The hydrodynamic size and zeta potential of GNPs and PGNPs was detected using dynamic light scattering (Malvern Nano ZS, Malvern, UK). GNPs or PGNPs at concentrations of 500 μg/mL in water or in medium with 10% FBS were used for size analysis. GNPs at a concentration of 2.5 nM was used for zeta potential analysis, and different pHs were selected in order to reveal the surface charge of the particles.

### Cellular uptake of PGNPs

1 mL of PGNPs (stock solution) was centrifuged in centrifugal filter (100,000 MWCO), washed with PBS (pH = 7.4), and dissolved in 1 mL of PBS before experiments. All cells were cultured in 12-well plate (50,000 cells/well) and treated with PGNPs at 2.5 nM for 0.5–72 hrs or at a concentration range (0.5, 1.0, 2.5, 5.0, 10.0, 20.0 nM) for 24 hrs. After incubation, samples including floating cells were harvested, and washed with PBS (1 mL × 3). The harvested cells were re-suspended in 500 μL of culture medium and cell density was counted. 200 μL of cell samples were incubated with 400 μL of Aqua Regia at 37°C for 24 hrs. The cell samples were centrifuged. 500 μL of supernatant was diluted to 10.0 mL in ultrapure water and used for ICP-MS measurements (Agilent 7500 series, Agilent Technologies, United States). A series of gold standard solutions (200, 100, 75, 50, 20, 10, 5 and 2 ppb) were prepared before measurements. The resulting calibration curve was used to calculate the gold content taken up by different cells.

### Transmission electron microscopy (TEM)

1 mL of PGNPs (stock solution) was centrifuged in centrifugal filter (100,000 MWCO), washed with PBS (pH = 7.4), and dissolved in 1 mL of PBS. Cells were treated with PGNPs (2.5 nM) for 24 hrs, and washed with PBS twice. The cells were harvested and washed with PBS twice. The clustered cells were fixed with 2.5% glutaraldehyde in 0.1 M of Sodium Cacodylate buffer (Tousimis Research Corporation) for 30 min at room temperature. The samples were washed with PBS again and sectioned. Ultrathin sections were examined using a JEOL 1200 EX transmission electron microscope (JEOL, Tokyo, Japan). The images were acquired using an AMT 2k CCD Camera.

### MDR cells screening

The establishments of MDR cell lines were performed by treating H460 cells with PTX or PGNPs of gradient concentrations (with the PTX concentrations of 10, 20, 40, 80, 100 nM) for 100 days, as reported previously [43]. And each concentration was administrated for two times. During each administration, cells were treated with PTX or PGNPs for continuous 3 days and then cultured in fresh medium for another 7 days. When it was necessary, cells were passaged for further culture. We obtained two cell lines in this experiment. PTX-treated cells named H460_PTX_ and PGNP-treated cells named H460_PGNP_.

### Pgp expression

After screening, the cells were harvested and lysed in RIPA lysis buffer (Beyotime, China) which contained 1% proteasome inhibitor (P2714, Sigma) and 1 mM phenyl methane sulfonyl fluoride (PMSF) to detect the expression of Pgp. Equal amounts (25 μg) of protein were loaded onto SDS-PAGE for separation and then transferred onto a PVDF membrane. The membrane was blocked with 5% w/v nonfat dry milk (in TBS with 0.05% Tween-20). After incubation with Mdr-1 Antibody (mouse monoclonal IgG2b, Santa Cruze, USA) (1:1000) at 4°C overnight, the membrane was washed three times with TBST (TBS with 0.05% Tween-20) solution. The membrane was then incubated with secondary antibody (1:5000) at room temperature for 1 h followed by three washes with TBST. The protein bands were developed by incubation with a luminescent reagent. ImageJ was used to quantify the band intensity.

### Pgp function assay

Pgp function in H460, H460_PTX_ and H460_PGNP_ were measured using a Rhodamine 123 (Rh123) assay. Cells were treated with Rh123 (2.5 μM) in the absence or presence of Pgp inhibitor Reversan (Life Technology, USA) at 10 μg/mL for 24 hrs. Then the cells were collected and washed with ice-cold PBS for three times in order to remove the adsorbed Rh123 on cell surface. The cells were re-suspended in PBS to the final cell concentration of 300,000/mL, placed in ice and analysed immediately on a Guava Easy Cyte Miniflow Cytometry System (Guava Millipore, Merck KGaA, Germany).

### Intracellular drug concentration

1 mL of f-PGNPs (stock solution) was centrifuged in centrifugal filter (100,000 MWCO), washed with PBS (pH = 7.4), and dissolved in 1 mL of PBS. For H460, cells were treated with fluorescence labeled PTX (f-PTX, 50 nM) or fluorescence labeled PGNPs (f-PGNPs, the total concentration of PTX was 50 nM) for 24 hrs and then the culture medium was replaced with fresh medium. After further incubation for 48 hrs, the cells were harvested and washed with PBS twice for flow cytometry analysis. For H460TaxR, cells were treated with f-PTX, f-PGNPs or f-PTX/Pgp inhibitor for continuous 72 hrs. After that, cells were harvested and washed with PBS twice for flow cytometry analysis.

### Cytotoxicity assay

The PGNPs in stock solution was washed with PBS in centrifugal filter (100,000 MWCO) at 4°C. The obtained PGNPs was dissolved in the same volume of PBS. Cells in 96-well plates were cultured with PTX, PGNPs or fresh culture medium for 72 hrs. Cell viability was determined by CellTiter-Glo® Luminescent Cell Viability Assay kit (Progema corporation, USA). The EC_50_ value was calculated using a four-parameter regression equations (Sigmaplot 12.0, Systat Software, Inc, UK).

### Fluorescent microscopy

1 mL of f-PGNPs (stock solution) was centrifuged in centrifugal filter (100,000 MWCO), washed with PBS (pH = 7.4), and dissolved in 1 mL of PBS. The cells were cultured in 24-well plates and treated with f-PTX (50 nM) and f-PGNPs (the concentration of PTX is 50 nM) for 24 hrs. After that, cells were washed with PBS twice and fixed in 4% paraformaldehyde (Alfa Asar) for 10 min at room temperature. Cells were then treated with 10% DAPI (Beyotime, China) in dark at 37°C for 5 min. The fluorescent pictures were obtained using a fluorescent microscope (OLYMPUS TH4-200 Olympus Optical Co Ltd, Tokyo, Japan).

## Results and Discussion

### Synthesis and characterization of PGNPs

**Fig 1:**
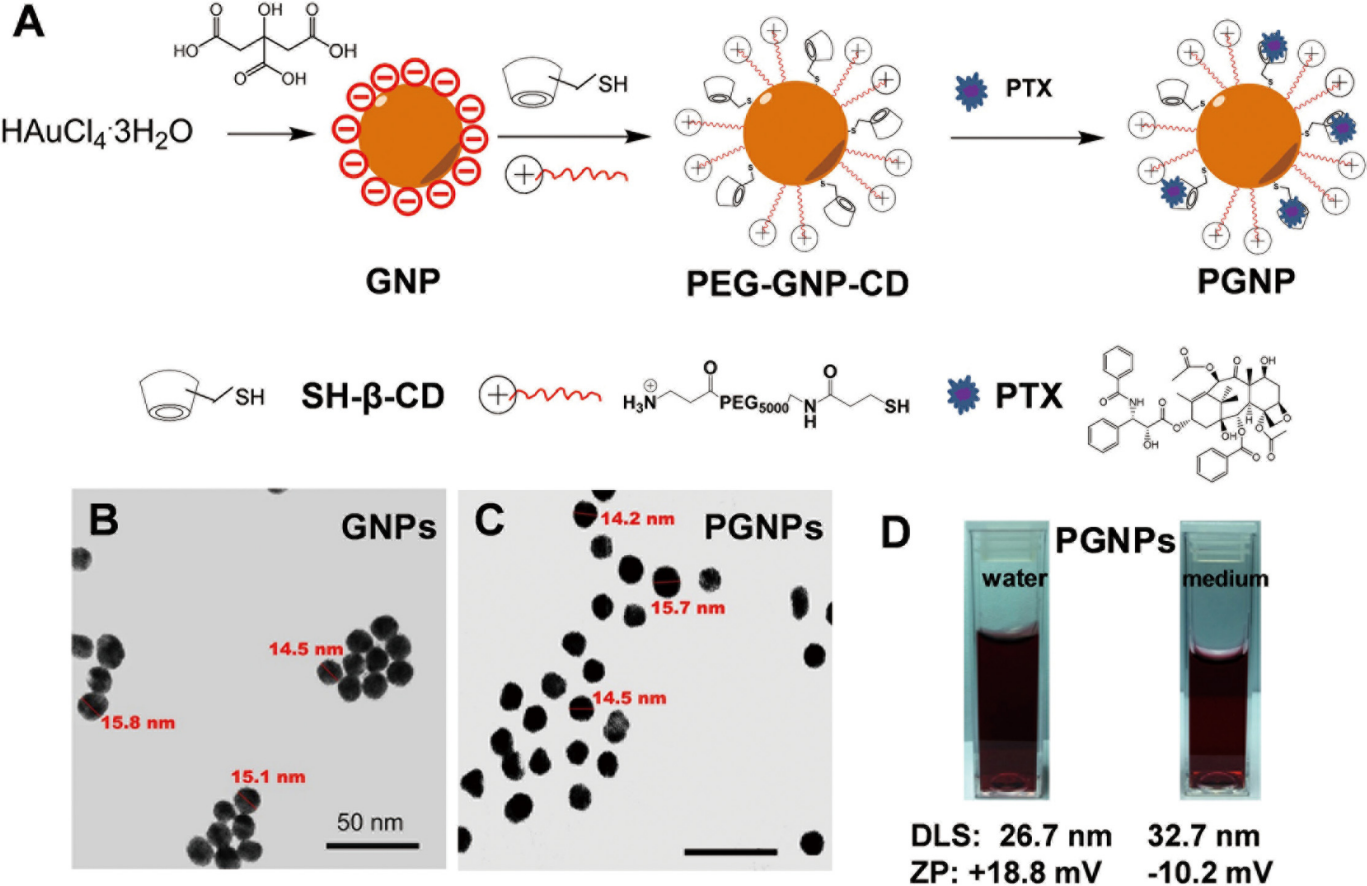
Preparation and characterization of PGNPs. (A) Preparation of PGNPs. (B, C) TEM images of GNPs and PGNPs. The scale bars are 50 nm. (D) Photographs showing that the PGNPs were well dispersed in water and in cell culture medium with 10% of FBS. The nanoparticles’ hydrodynamic size and zeta potential (ZP) are also shown.

To assemble a nanoconjugate with slow drug release, we attached β-cyclodextrin (β-CD) with thiol functional groups, and PEG_5000_ with amino functional groups to the GNPs to provide a drug-carrying moiety, as named PEG-GNP-CD (Fig 1A). The PEG_5000_ can not only enhance the water solubility of GNPs, but also increase the cell affinity due to its positive charge [44–46]. Drug molecules, such as PTX, are trapped in the hydrophobic pocket of β-CD through host-guest interactions and are then slowly released, likely through competitive host-guest interactions between β-CD and many cellular molecules, such as amino acids and sugar [42, 47–49].

After nanoconjugates synthesis and loading of PTX molecules, we thoroughly characterized the final product, PTX-loaded GNPs (PGNPs), using an array of analytical methods. Transmission electron microscopy (TEM) image showed that the average GNP core diameter was 15 nm with small aggregations (Fig 1B). After conjugation with PEG_5000_ and β-CD and loading of PTX, PGNPs showed similar GNP core diameters as 15 nm, but the water solubility was largely improved as determined by TEM (Fig 1C). Because the TEM only captured images of the metal core, we further determined the dynamic diameter of PGNPs in solution using the dynamic light-scattering (DLS) method. The pictures in Fig 1D indicated PGNPs had good aqueous solubility in both water and cell culture medium with 10% of fetal bovine serum (FBS). The hydrodynamic size of each PGNP was ~27 ± 5.3 nm in water. In cell culture medium with 10% FBS, the hydrodynamic size increased to ~33 ± 6.0 nm, indicating protein adsorption on PGNPs. Furthermore, the zeta potential of both GNPs and PGNPs was changed at different pHs (S1 Fig). The zeta potential of PGNPs was determined to be +18 mV in water as expected, indicating a positive surface charge. However, the zeta potential of PGNPs changed to -10 mV in cell culture medium with 10% FBS, which confirmed the protein adsorption on PGNPs (Fig 1D). Additionally, there were in average 28 PTX molecules on each nanoparticle, as determined by high performance liquid chromatography-mass spectrometry (HPLC) analysis.

### Loading and release profiles of PGNPs

**Fig 2:**
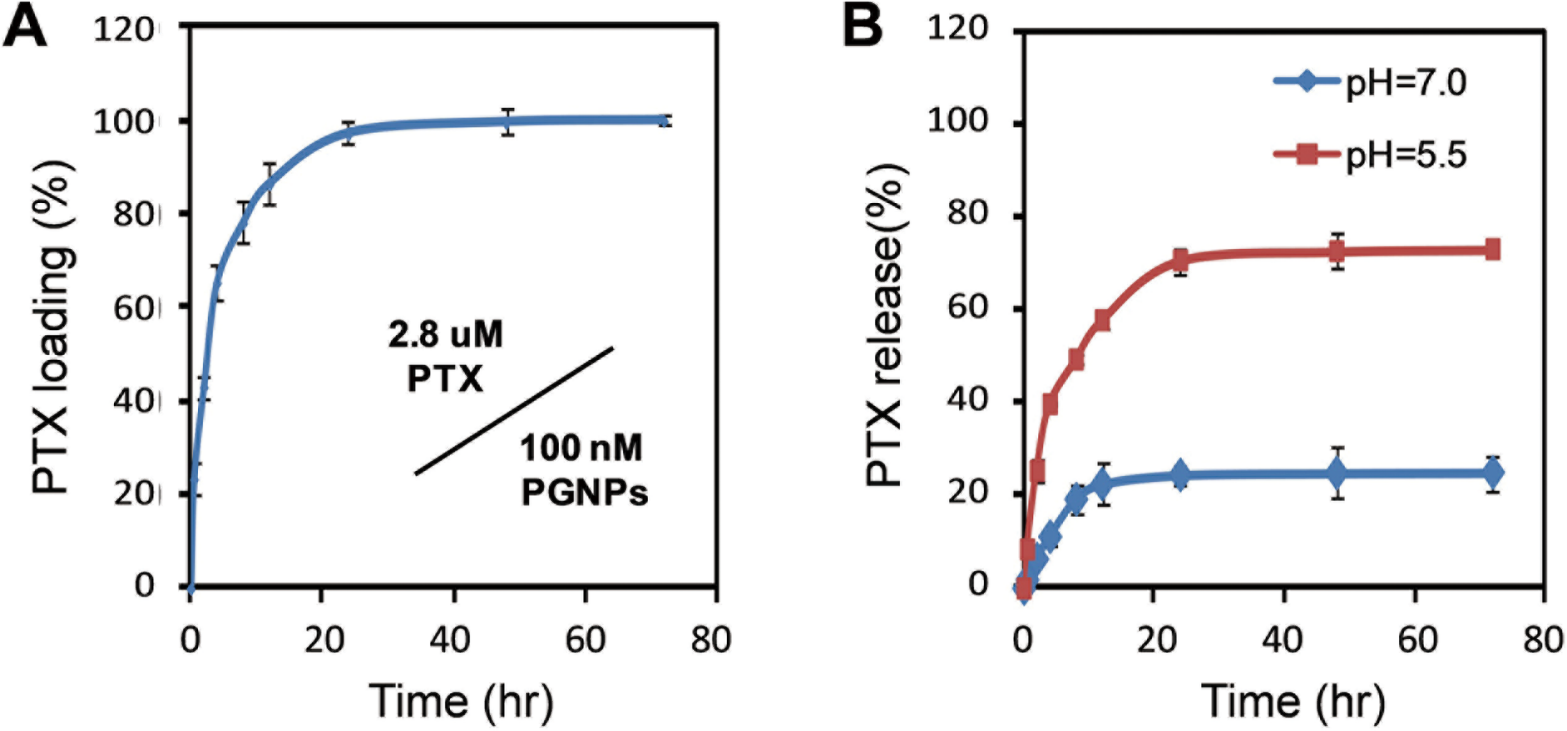
Loading and release profiles of PTX molecules on PGNPs. (A) Encapsulation of PTX onto GNPs with different incubation time. (B) Drug release from PGNPs at different pHs. Each data point was measured in triplicate. Data are mean±s.d.

The loading and release of the PTX on the PGNPs were also investigated by HPLC (Materials and Methods). PGNPs were prepared by adding 300 μL of PTX (0.4 mM) into 30 mL of PEG-GNP-CD solution (100 nM). The mixture was shaked at room temperature for different time. The equilibrium was reached after 24 hrs incubation (Fig 2A). Approximately 2.8 μM PTX was loaded to GNPs at equilibrium for 24 hrs, indicating a loading of 70%. The PTX release profile was then studied at pH 7.0 and 5.5. At pH 7.0, only 25% of the PTX molecules were spontaneously released from the PGNPs within 15 hrs. However, approximately 80% of the PTX molecules were released within the same time period at pH 5.5, suggesting that the drug molecules could more easily escape the nanoconjugates when located within endosomes or lysosomes inside cancer cells (Fig 2B).

### The suppression of drug resistance in H460 cells by PGNPs

**Fig 3:**
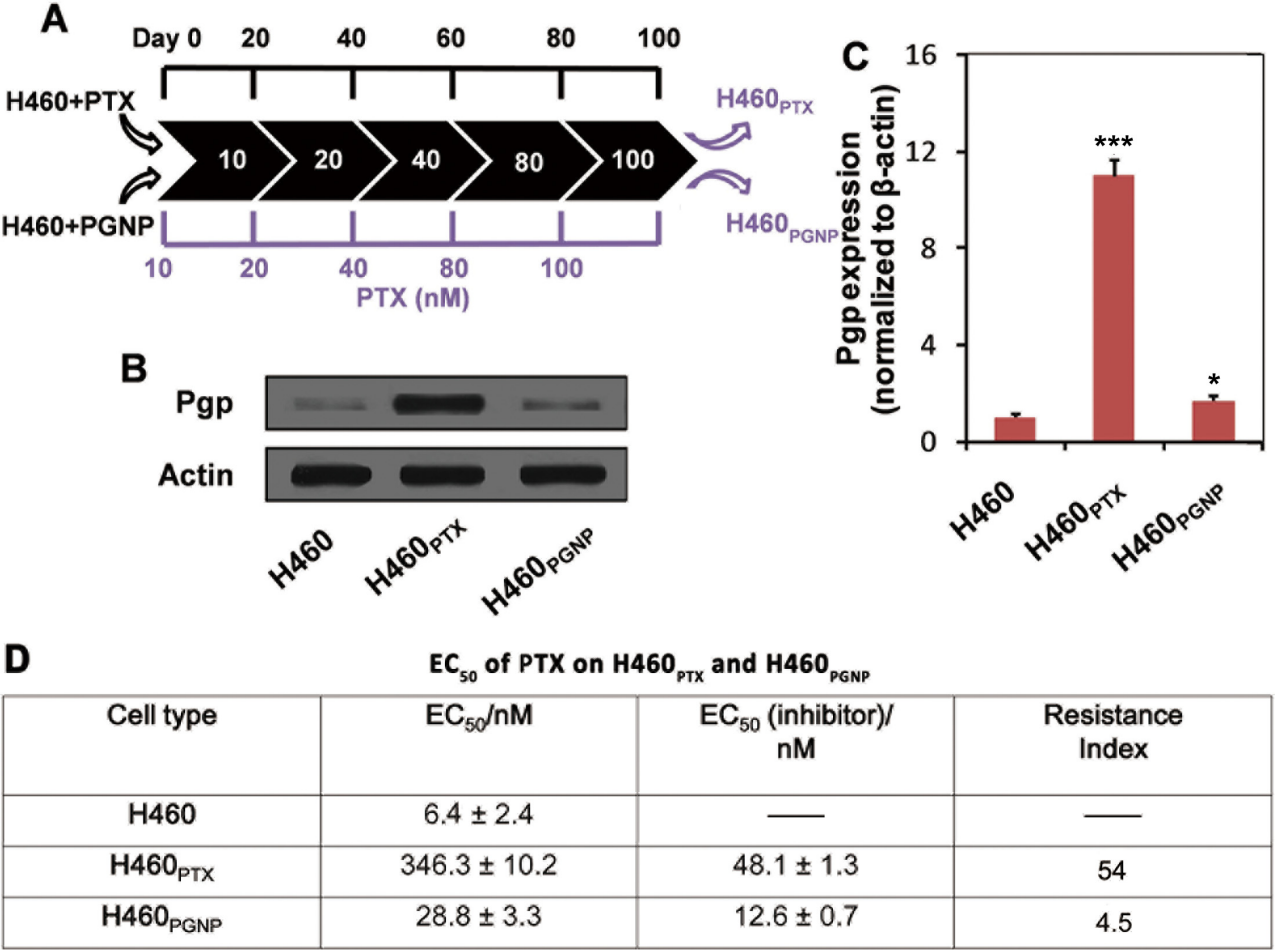
PGNPs block Pgp overexpression in durg-sensitive H460 cells. (A) Experimental scheme for 100-day treatment of cells with PTX or PGNPs with progressively increasing the concentration of PTX. (B) Pgp expression in H460, H460_PTX_, and H460_PGNP_ cells. (C) Quantification of Pgp band intensities in (B), as determined by ImageJ. *P<0.05, compared with that of H460 under the same condition; ***P<0.001, compared with that of H460 under the same condition. (D) EC_50_ of PTX on H460_PTX_ and H460_PGNP_. EC_50_ (inhibitor), EC_50_ of PTX on H460_PTX_ and H460_PGNP_ in the presence of a Pgp inhibitor (Reversan). Each experiment was repeated at least three times. Data are mean±s.d.

Considering that lung cancer is still the deadliest cancer [50] and the development of severe drug resistance during chemotherapy as one of the key roadblocks to its treatment [51], the NSCLC cell line H460 was used in our study. Pgp overexpression always occurs after a period of chemotherapy. During this process, a higher drug dose always induces more drug resistance [52]. To investigate this drug resistance induction, we incubated drug-sensitive H460 cells with PTX or PGNPs at the equivalent drug concentration, which was progressively increased from 10 nM to 100 nM over the course of 100 days (Fig 3A; see the Experimental section for further details). The resulting H460 sub-lines H460_PTX_ (treated with PTX) and H460_PGNP_ (treated with PGNPs) were characterized for their Pgp expression levels and their sensitivity to PTX treatment. Compared with the parent H460 cells, H460_PGNP_ exhibited an increased Pgp expression level (Fig 3B and 3C) and EC_50_ value of PTX on H460_PGNP_ cells was 28.8 nM (Fig 3D), indicating the acquirement of a slight drug resistance. By contrast, H460_PTX_ exhibited a significant increase in Pgp expression level (Fig 3B and 3C) and EC_50_ value of PTX on H460_PTX_ was 346.3 nM (Fig 3D), which was 54-fold higher than that of H460, indicating that much higher resistance was induced by PTX.

**Fig 4:**
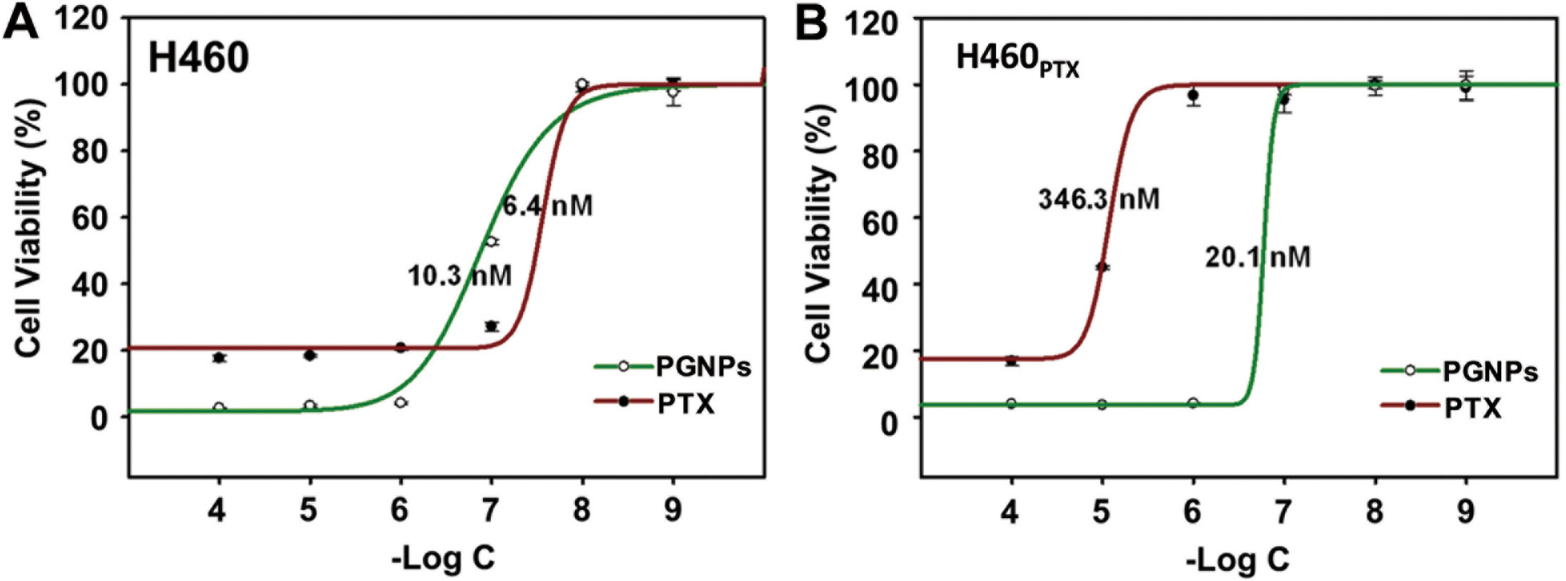
PTX- or PGNP-induced cytotoxicity in H460 and H460_PTX_ cells. The PTX concentration was determined by HPLC-MS. Cells were incubated with PTX or PGNPs for 72 hrs and cell viability was determined by CellTiter-Glo® Luminescent Cell Viability Assay. Each data point was measured in triplicate. Data are mean±s.d.

The cytotoxicity of PTX and PGNPs in H460 cells and H460_PTX_ was also examined. In H460 cells, the cytotoxicity of PGNPs was not so different from that of PTX (EC_50_ of 6.4 and 10.3 nM, Fig 4A). However, in drug-resistant H460_PTX_ cells, PGNPs was much more potent compared to PTX with a 17-fold lower EC_50_ value (EC_50_ 20.1 vs 346.3 nM, Fig 4B), indicating that PGNPs were able to avoid being pumped out of cells by Pgp. This result is consistent with previous works, which showed the enhanced anticancer activity of PTX when conjugated to GNPs. Chen et al. showed that cancer-targeting GNPs loaded with PTX exhibited a better anticancer activity in human ovarian cancer cell line SKOV-3 compared to free PTX [53]. However, its anticancer activity in drug resistance cells was not studied. PTX has a IC_50_ value above 1 μM in resistant MES-SA/Dx5 cells, whereas GNPs loaded with PTX molecules through DNA linkers exhibited an IC_50_ value of 104.5 nM [39].

**Fig 5:**
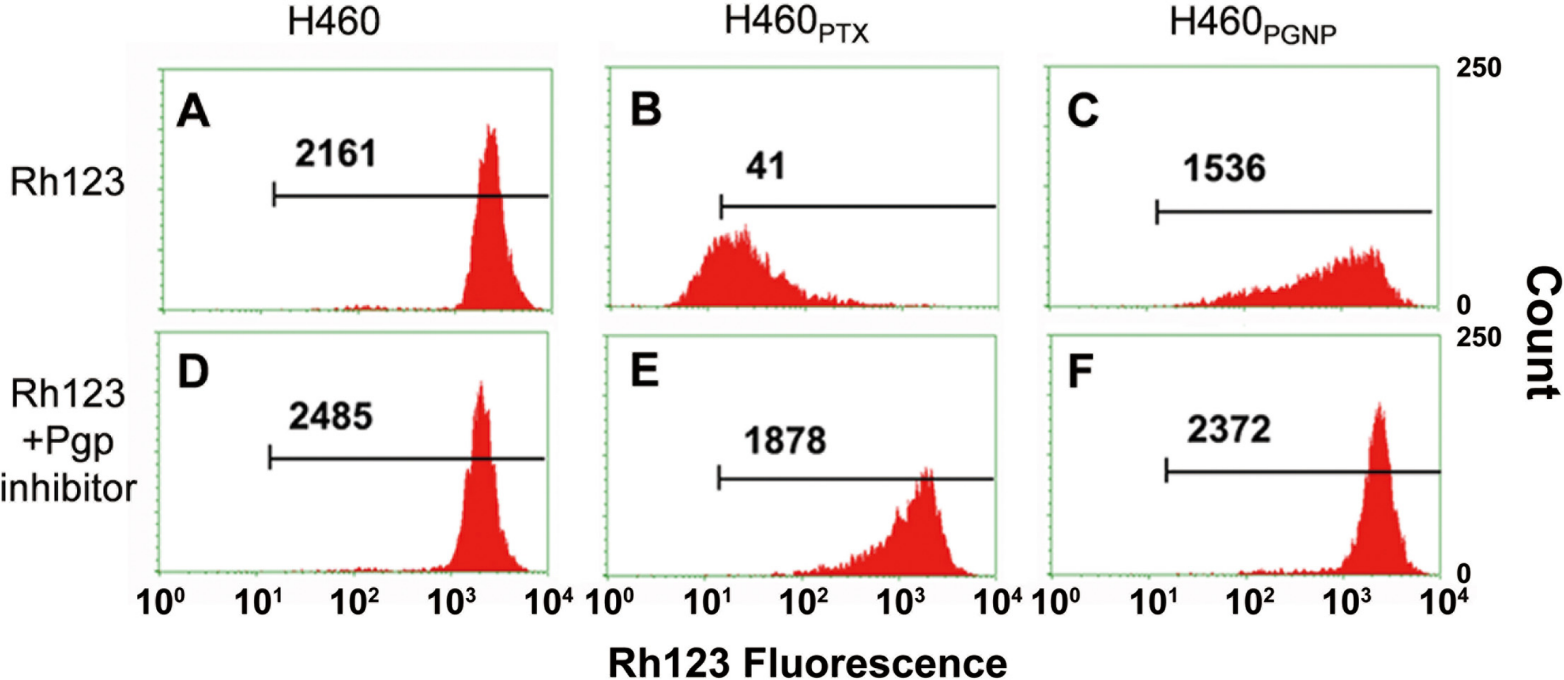
Pgp function determined by intracellular Rhodamine123 (Rh123) accumulation in different cell lines. Cells were treated with 2.5 μM Rh123 with or without adding Pgp inhibitor. The intracellular accumulation of Rh123 was determined by flow cytometry. Numbers in plots are average fluorescence intensities of intracellular Rh123. The figure is the representative of results from three independent experiments.

Pgp function in H460_PGNP_ and H460_PTX_ was also examined using a standard Rhodamine 123 (Rh123) efflux assay (Fig 5) [54] After incubation with Rh123 with different cells for 24 hrs, the cellular uptake of Rh123 was determined by flow cytometry. High cellular uptake of Rh123 was found in H460 (Fig 5A) and H460_PGNP_ (Fig 5C) cells, with only limited amount of Rh123 found in H460_PTX_ cells (Fig 5B). The results show that H460_PTX_ cells pump Rh123 molecules out at a much higher rate than in H460_PGNP_ cells. To confirm the low cellular uptake of Rh123 in H460_PTX_ was caused by high Pgp expression, a Pgp inhibitor, Reversan, was incubated with cells for 4 hrs before incubation with Rh123. A much higher accumulation of Rh123 in H460_PTX_ cells (Fig 5E) was found, with no clear differences in H460 and H460_PGNP_ cells (Fig 5D and 5F).

Chemotherapy-induced Pgp overexpression is an important self-protection mechanism for cancer cells. Although the underlying mechanism is of great complexity and is not yet fully understood, investigations have suggested that in cancer cells, the MDR-1 gene is activated by anti-cancer drug treatments [3, 55]. With increasing drug doses, Pgp overexpression and drug resistance are further exacerbated. As a result, Pgp-overexpressing cancer cells can no longer be killed by conventional chemotherapy. Sudden exposure to a high drug concentration is a key factor for inducing Pgp overexpression [52, 56, 57], and regular intermittent drug administration may give emerging drug-resistant cells time to survive and proliferate. Recent studies have thus shown that continuous low-dose drug treatment is somewhat helpful for partially avoiding Pgp overexpression [58, 59]. In the current study, the nanoconjugate PGNPs showed sustained slow drug release, and the lower intracellular drug accumulation generated by the PGNPs compared with free PTX treatment likely explains the lower Pgp overexpression observed during the long-term drug treatment.

### The binding and internalization of PGNPs by H460 and H460_PTX_ cells

**Fig 6:**
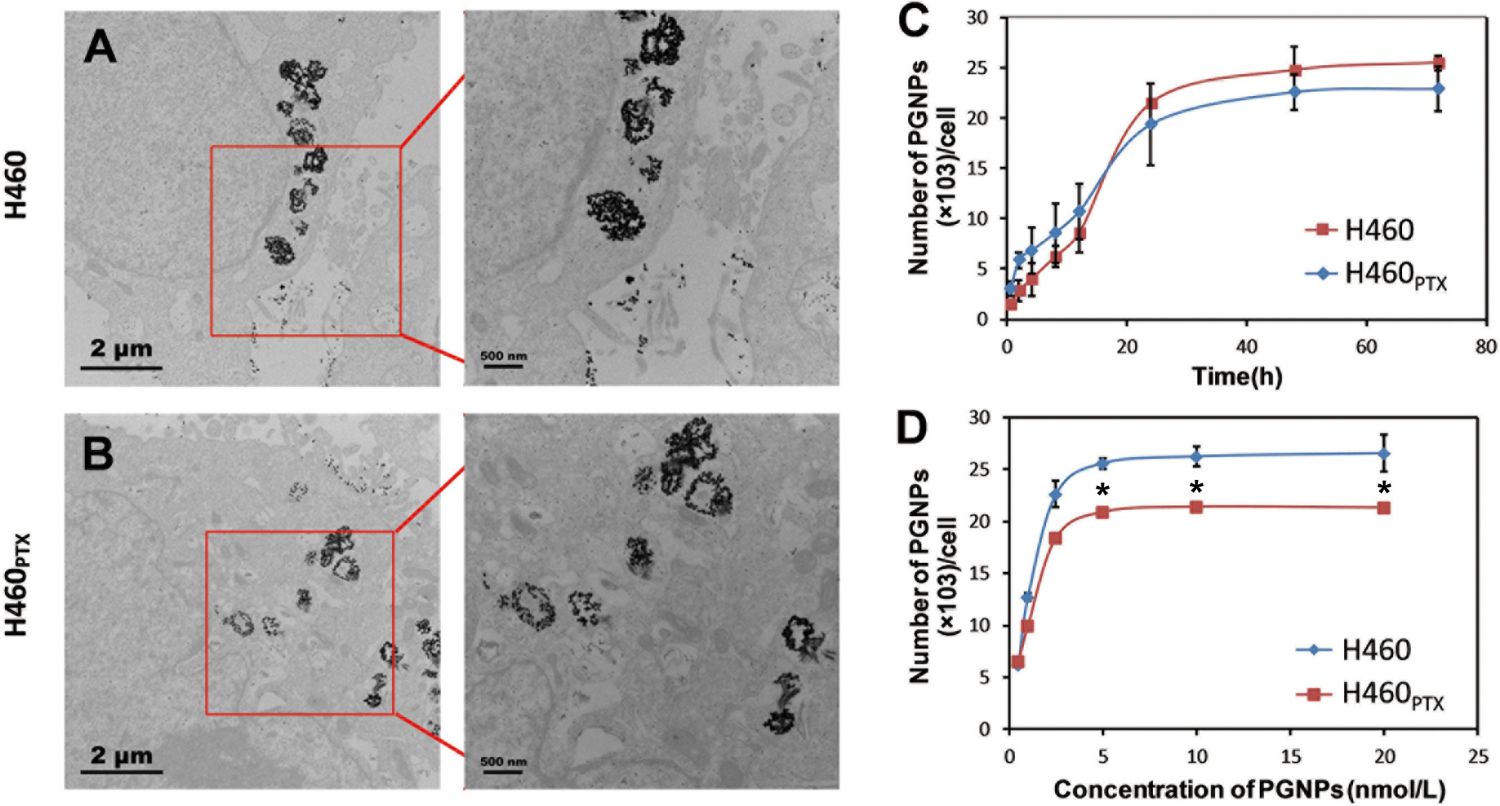
Internalization of PGNPs by H460 and H460_PTX_ Cells. (A, B) TEM images of PGNPs internalized by H460 and H460_PTX_ cells. The figure is the representive of results from three independent expeiments. (C) Time-dependent cellular uptake of PGNPs by H460 and H460_PTX_ cells. Cells were treated with PGNPs at 2.5 nM for indicated times and cellular uptake of PGNPs was determined by ICP-MS. (D) Dose-dependent uptake of PGNPs by H460 and H460_PTX_ cells after treatment for 24 hrs. Each data point was measured in triplicate. Data are mean±s.d. *P<0.05, compared with that of H460 under the same treatment.

With the Pgp high-expressed H460_PTX_ cells in hand, we were able to investigate the cellular uptake of PGNPs in drug resistant cancer cells and if PGNPs could evade Pgp-induced drug efflux to achieve potent cancer cell death in drug-resistant cells. The internalization of PGNPs by H460 and H460_PTX_ cells was first examined by visualizing cell cross-sections using TEM. The TEM images showed that the nanoparticles entered both H460 and H460_PTX_ cells and were mainly present in vesicle-like endosomes and lysosomes (Fig 6A and 6B). The Au content in the cells was then quantitatively analyzed by inductively coupled plasma-mass spectrometry (ICP-MS). When the cells were treated with PGNPs at 2.5 nM, the cellular Au content increased with time until reaching a plateau in approximately 24 hrs. Approximately 24,000 and 26,000 GNPs entered each H460 and H460_PTX_ cell, respectively (Fig 6C). PGNPs showed similar cellular uptake rate in H460 and H460_PTX_ cells, indicating a negative correlation between the cell binding and internalization of PGNPs and Pgp expression. We then fixed the PGNP incubation time as 24 hrs and cells were incubated with PGNPs at various concentrations, the maximal internalization of PGNPs was achieved at a PGNP concentration of 5 nM for both H460 and H460_PTX_ cells (Fig 6D), with slight higher PGNP internalization in H460 cells than H460_PTX_ cells.

At 4°C, such internalization was heavily inhibited, suggesting that the PGNPs internalization was energy dependent (S2 Fig). A GTPase inhibitor, NaN_3_, also inhibited PGNP internalization. These data demonstrated that the PGNPs were poor Pgp substrates and were internalized through endocytosis (S2 Fig) through which PGNPs effectively delivered drugs into cytoplasm, causing cancer cell death and lower expression of P-gp. Based on the amount of PGNPs in each cell and the original PTX loading of the PGNPs, we calculated the approximate amount of PTX in each cell. Under the equilibrium condition, approximately 1.12×10^−18^ or 1.21×10^−18^ moles of PTX molecules entered H460 and H460_PTX_ cancer cells, respectively, of which approximately 0.92×10^−18^ or 1.0×10^−18^ moles could be released from nanoparticles in 25 hrs as determined based on PTX release profile.

### PTX accumulation in H460 and H460_PTX_ cells

**Fig 7:**
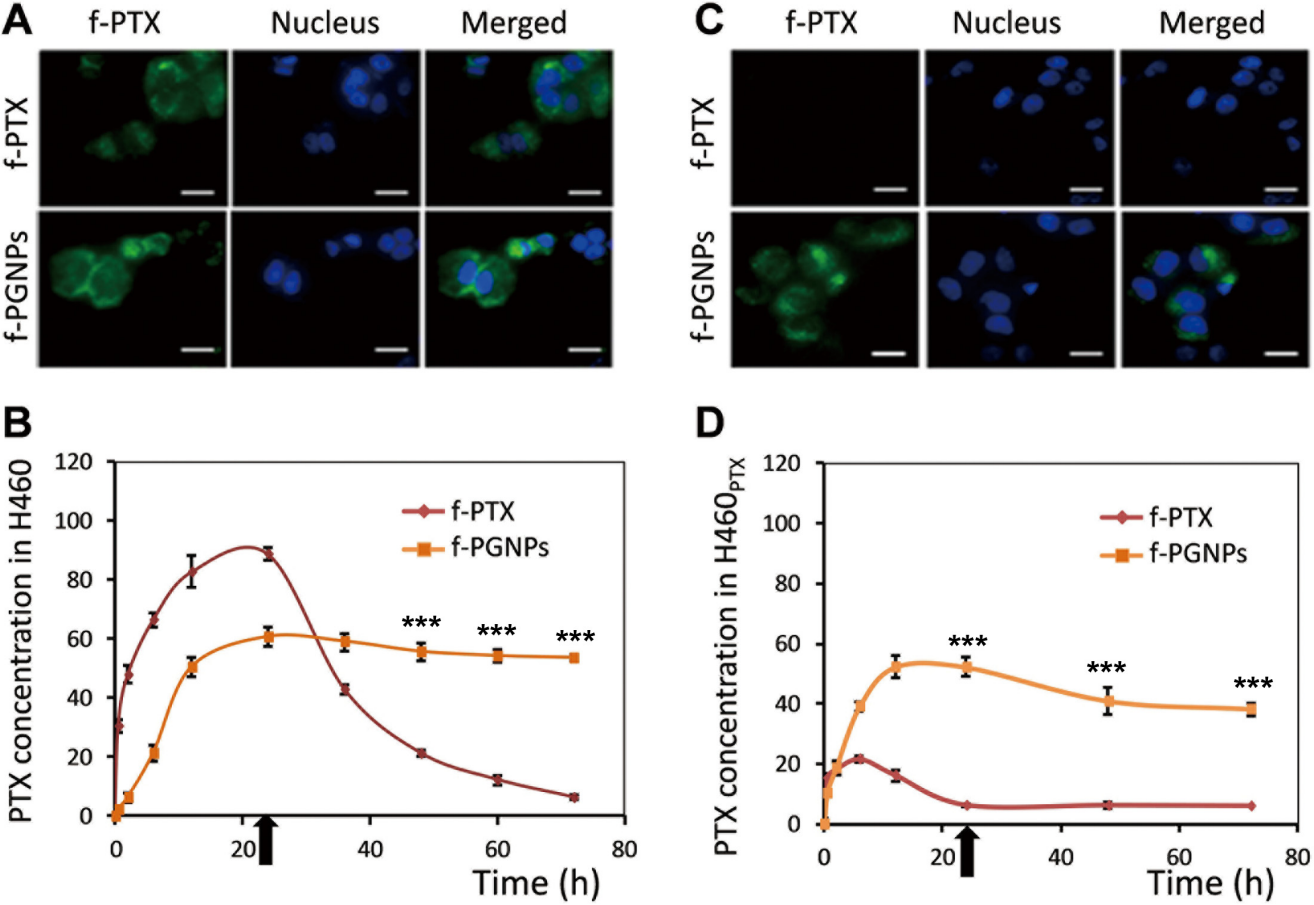
Cellular drug accumulation of f-PTX and f-PGNPs. Fluorescence images of (A) drug-sensitive H460 cells and (C) drug-resistant H460_PTX_ cells treated with f-PTX and f-PGNPs. H460 and H460_PTX_ cells were treated with free f-PTX (50 nM) or f-PGNP (the total concentration of f-PTX was 50 nM) for 24 hrs and the photos were captured by fluorescent microscopy. The scale bars are 10 μm. The figure is the representive of results from three independent expeiments. Intracellular accumulation of f-PTX in (B) drug-sensitive H460 cells and (D) drug-resistant H460_PTX_ cells treated with f-PTX or f-PGNPs for different times, as measured by flow cytometry. Black arrows indicate removal of free f-PTX and f-PGNPs at 24 hrs of incubation. Each data point was measured in triplicate. Data are mean±s.d. ***P<0.001 compared with that treated with f-PTX for the same time period.

Aside from drug-induced Pgp overexpression during long-term drug treatment of drug-sensitive cells, one form of direct resistance to conventional chemotherapy in drug-resistant cells is quick loss of drug accumulation as a result of Pgp-driven drug efflux. Thus, we next tested whether the nanodrug could evade drug efflux and maintain the cellular drug concentration and drug efficacy in drug-resistant cancer cells. We first used fluorescently labeled PTX (f-PTX) to test this hypothesis in drug-sensitive H460 and drug-resistant H460_PTX_ cancer cells. Both f-PTX and fluorescence labeled PGNPs (f-PGNP) molecules maintained general cytotoxicity (S3 Fig) and were highly fluorescent. When drug-sensitive H460 cells were treated with f-PTX or f-PGNPs at equivalent PTX concentration of 50 nM, the cells quickly became fluorescent as drug molecules accumulated inside them (Fig 7A).

We then monitored the accumulation of f-PTX with time by checking the fluorescence intensity by flow cytometry. f-PTX and f-PGNPs showed similar cellular accumulation in drug-sensitive H460 cells, with maximal PTX accumulation at 24 hrs of incubation (Fig 7B), which was consistent with the cellular uptake of PGNPs. After the free PTX and f-PGNPs were removed from the cell culture medium at 24 hrs of treatment, the cellular drug concentration in the f-PTX-treated cells was reduced to nearly zero within 48 hrs (Fig 7B). In contrast, a high drug concentration was maintained with f-PGNP treatment for up to 72 hrs (Fig 7B). These results showed that when cancer cells were treated with small molecular drugs, the cellular drug concentration was dynamic and could only be maintained at a high level for a short time because of the strong cell penetrating of the small molecular drugs. Meanwhile, a high drug level could be maintained over the entire experimental period with the nanoconjugate delivery system, probably due to the low clean-up rate of nanoparticles by cancer cells. These contrasting accumulation behaviors did not appear to affect the therapeutic effect of the drug in H460 cells (Fig 3).

In Pgp-overexpressing H460_PTX_ cells, f-PTX molecules were quickly and efficiently pumped out, resulting in low cellular drug concentration throughout the treatment, as showing in fluorescent microscopy (Fig 7C). Conversely, f-PGNPs successfully evaded Pgp pumps, and the cellular PTX accumulation was much higher than that of f-PTX, at a concentration that was approximately 4-fold higher than that after free f-PTX treatment for 24 hrs (Fig 7D). After removal of free f-PGNPs at 24 hrs of treatment, the cellular PTX concentration was maintained with f-PGNP treatment for up to 72 hrs, with approximately 7-fold higher than that after free f-PTX treatment. The different PTX concentrations in H460_PTX_ cells after treatment with f-PTX and f-PGNPs resulted in drastically different cytotoxicities. These results demonstrated that the nanoparticle-delivered drug was able to evade Pgp-mediated drug resistance and to remain highly cytotoxic to drug-resistant cancer cells. There are several tentative explanations as to the result. First, in contrast to free drug, nanoparticle-delivered PTX molecules are mostly found in endosomes or lysosomes (Fig 6A and 6B), with the nanoconjugates located away from the membrane-bound Pgp protein. Second, even if PGNPs leak into the cytoplasm, Pgp is unlikely to bind the nanoparticles efficiently and to pump them out of the cell because the nanoparticles do not fit into the binding pockets of the Pgp protein. Third, even though PTX molecules are released into the cytoplasm, there is still strong competition between binding to tubulin molecules and moving to the cell membrane to bind Pgp protein.

## Conclusion

**Fig 8:**
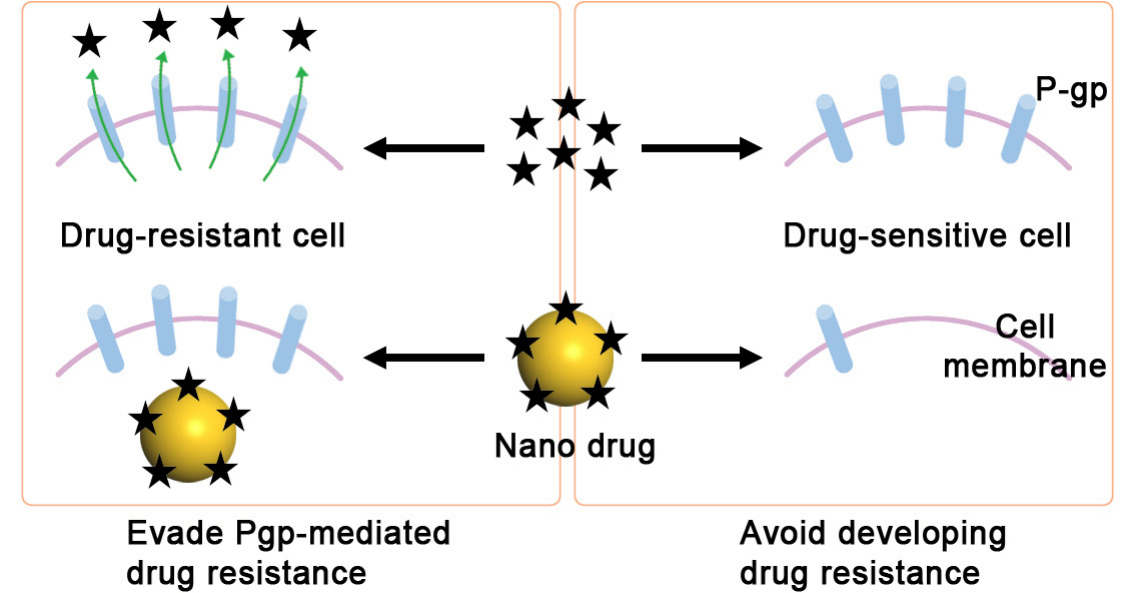
A working model showing the different effects on cancer cells produced by free PTX and PGNPs.

In conclusion, taking advantage of the ease of cell internalization and the slow drug release of PGNP nanoconjugates, we effectively prevented Pgp overexpression in drug-sensitive cancer cells after long-term treatment, indicating the possibility of long-term or repeated nanodrug use without induction of drug resistance. Furthermore, the nanodrug successfully avoids being pumped out of cells by Pgp protein in Pgp-overexpressing drug-resistant cancer cells, allowing the nanodrug to maintain its high cytotoxicity (Fig 8). Our findings from this investigation could be a general strategy against drug resistant cancers because the slow drug release and cytoplasm drug delivery could be realized using other nano vehicles and similar cytotoxicity could be induced in other cancer cells. Although significant research efforts are needed, we see a promising future of this approach.

## Supporting Information

S1 Fig Zeta potential of GNPs and PGNPs at different pHs. Nanoparticle concentrations were 2.5 nM and culture medium contained 10% of FBS. Each data point was measured in triplicate. Data are mean±s.d.
(TIF)

S2 Fig Endocytosis of PGNPs in cancer cells. (a) Cellular uptake of PGNP (2.5 nM) at 4°C, 37°C or at 37°C with NaN_3_ (10 mM) in H460 cells. (b) Cellular uptake of PGNP (2.5 nM) at 4°C, 37°C or at 37°C with NaN_3_ (10 mM) in H460_PTX_ cells. Each experiment was repeated at least three times. Data are mean±s.d. ***P<0.001, compared with that treated at 37°C for the same time period.
(TIF)

S3 Fig Comparison of cytotoxicity of PTX *vs* f-PTX and PGNP *vs* f-PGNP. Cells were treated with PTX, f-PTX, PGNP, f-PGNP, or GNP for 72 hrs and the cell viabilities were determined by CellTiter-Glo® Luminescent Cell Viability Assay. Each experiment was repeated at least three times. Data are mean±s.d.
(TIF)
