# Supplementary figures and images for: Reducing Both Pgp Overexpression and Drug Efflux with Anti-Cancer Gold-Paclitaxel Nanoconjugates
Source: PLoS One. 2016 Jul 28;11(7):e0160042. doi: 10.1371/journal.pone.0160042 (PMC4965149; doi:10.1371/journal.pone.0160042)

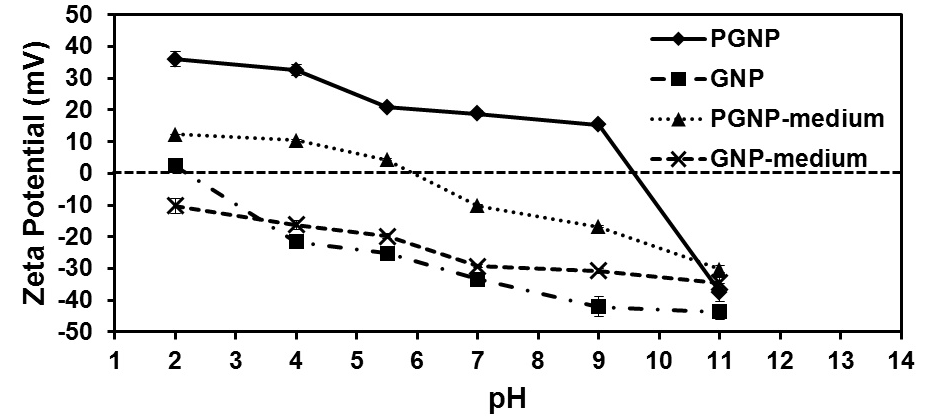

Supplement: S1 Fig — Nanoparticle concentrations were 2.5 nM and culture medium contained 10% of FBS. Each data point was measured in triplicate. Data are mean±s.d. (TIF) [file pone.0160042.s001.tif]

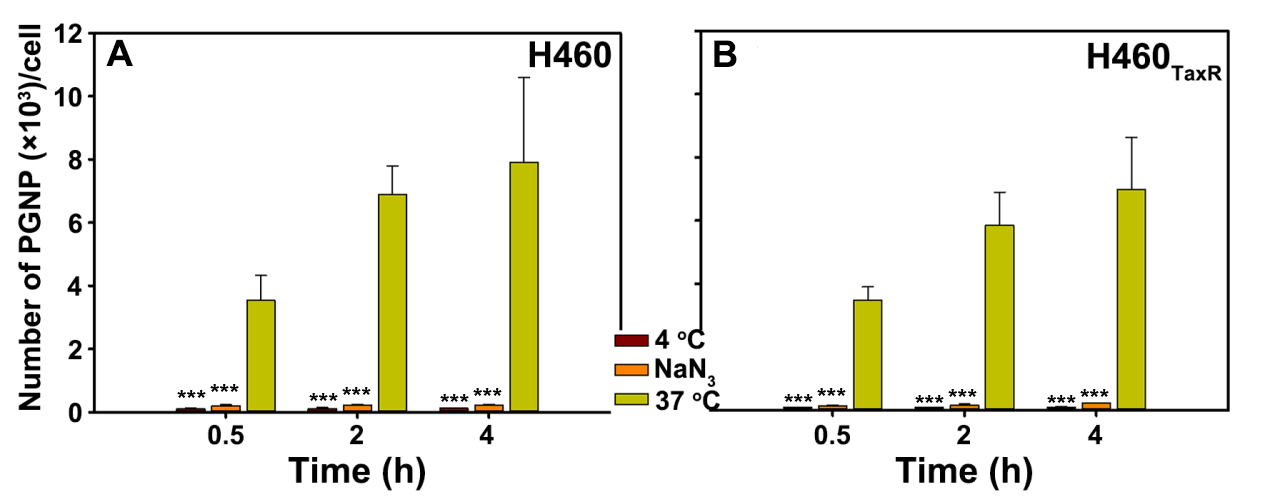

Supplement: S2 Fig — (a) Cellular uptake of PGNP (2.5 nM) at 4°C, 37°C or at 37°C with NaN3 (10 mM) in H460 cells. (b) Cellular uptake of PGNP (2.5 nM) at 4°C, 37°C or at 37°C with NaN3 (10 mM) in H460PTX cells. Each experiment was repeated at least three times. Data are mean±s.d. ***P<0.001, compared with that treated at 37°C for the same time period. (TIF) [file pone.0160042.s002.tif]

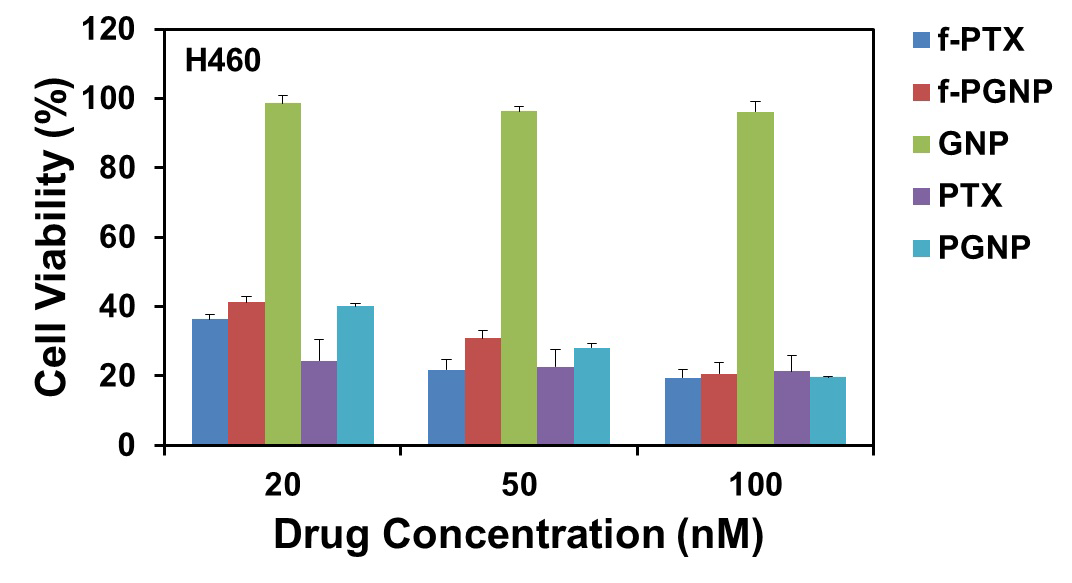

Supplement: S3 Fig — Cells were treated with PTX, f-PTX, PGNP, f-PGNP, or GNP for 72 hrs and the cell viabilities were determined by CellTiter-Glo® Luminescent Cell Viability Assay. Each experiment was repeated at least three times. Data are mean±s.d. (TIF) [file pone.0160042.s003.tif]
